# Coupling Between Carbon and Nitrogen Metabolic Processes Mediated by Coastal Microbes in *Synechococcus*-Derived Organic Matter Addition Incubations

**DOI:** 10.3389/fmicb.2020.01041

**Published:** 2020-05-25

**Authors:** Rui Xie, Yu Wang, Qi Chen, Weidong Guo, Nianzhi Jiao, Qiang Zheng

**Affiliations:** ^1^State Key Laboratory of Marine Environmental Science, College of Ocean and Earth Sciences, Institute of Marine Microbes and Ecospheres, Xiamen University, Xiamen, China; ^2^Fujian Key Laboratory of Marine Carbon Sequestration, Xiamen University, Xiamen, China; ^3^College of Environment and Ecology, Xiamen University, Xiamen, China; ^4^Key Laboratory of Coastal and Wetland Ecosystems, Ministry of Education, Xiamen University, Xiamen, China

**Keywords:** heterotrophic bacteria, *Synechococcus*-derived organic matter, nitrogen cycling, fluorescent dissolved organic matter, recalcitrant dissolved organic matter

## Abstract

Phytoplankton are major contributors to labile organic matter in the upper ocean. Diverse heterotrophic bacteria successively metabolize these labile compounds and drive elemental biogeochemical cycling. We investigated the bioavailability of *Synechococcus*-derived organic matter (SOM) by estuarine and coastal microbes during 180-day dark incubations. Variations in organic carbon, inorganic nutrients, fluorescent dissolved organic matter (FDOM), and total/active microbial communities were monitored. The entire incubations could be partitioned into three phases (labeled I, II, and III) based on the total organic carbon (TOC) consumption rates of 6.38–7.01, 0.53–0.64, and 0.10–0.13 μmol C L^–1^ day^–1^, respectively. This corresponded with accumulation processes of NH_4_^+^, NO_2_^–^, and NO_3_^–^, respectively. One tryptophan-like (C1) and three humic-like (C2, C3, and C4) FDOM components were identified. The intensity variation of C1 followed bacterial growth activities, and C2, C3, and C4 displayed labile, semi-labile, and refractory DOM characteristics, respectively. Alphaproteobacteria, Gammaproteobacteria, Bacteroidetes, and Actinobacteria dominated the quickly consumed process of SOM (phase I) coupled with a substantial amount of NH_4_^+^ generation. Thaumarchaeota became an abundant population with the highest activities in phase II, especially in the free-living size-fraction, and these organisms could perform chemoautotroph processes through the ammonia oxidation. Microbial populations frequently found in the dark ocean, even the deep sea, became abundant during phase III, in which Nitrospinae/Nitrospirae obtained energy through nitrite oxidation. Our results shed light on the transformation of different biological availability of organic carbon by coastal microorganisms which coupled with the regeneration of different form of inorganic nitrogen.

## Introduction

Marine phytoplankton contribute to approximately one half of the global net primary production through photosynthesis (Field et al., 1998). Phytoplankton release organic matter into surrounding environments in the form of dissolved organic matter (DOM) and particulate organic matter (POM) through various pathways, including active/passive secretion of photosynthate, zooplankton grazing, viral lysis, and aging phytoplankton cells decay (Muhlenbruch et al., 2018). A significant fraction of this organic matter is channeled via heterotrophic bacteria into the microbial loop (Azam, 1998). Heterotrophic bacteria ultimately determine the fate of this organic matter in the ocean, incorporating it into biomass (bacterial secondary production), respiring it back to CO_2_, or transforming the labile fraction into refractory fractions (Azam et al., 1983; Kirchman et al., 1991; Jiao et al., 2010; Sarmento et al., 2016). During these processes, heterotrophic bacteria also drive the elemental cycles of nitrogen (N) and phosphorus (P) in the ocean (Dyhrman et al., 2007; Falkowski et al., 2008; Hutchins and Fu, 2017).

Phytoplankton blooms are noticeable features of biological variability responding to changing physiochemical factors (e.g., N/P nutrient enrichment and temperature) in shallow estuarine and nearshore coastal ecosystems (Cloern, 1996; Teeling et al., 2012; Paerl and Otten, 2013). Moreover, unbalanced nutrient ratios are responsible for some non-diatom blooms as anthropogenic activities increase the amount of bioavailable inorganic N and P relative to Si (Conley and Johnstone, 1995; Justic et al., 1995; Cloern, 2001; Glibert and Burford, 2017). Rapid growth and accumulation of phytoplankton biomass during blooms require the transformation of a large number of inorganic nutrients into organic forms, which directly leads to marked changes in surrounding environmental conditions including depleted N/P inorganic nutrient levels, removal of CO_2_, and super-saturation of O_2_ (Cloern, 1996; Mahadevan et al., 2012).

Phytoplankton are also an important source of sinking particles in the ocean and contribute to the biological pump (the process by which carbon is exported from the euphotic zone to the dark ocean) (Durkin et al., 2016). A fraction of phytoplankton biomass, in the form of POM, is exported from surface water into the deep ocean (Richardson, 2019), especially in declining periods of phytoplankton blooms (Smetacek et al., 2012). However, the majority of POM is re-mineralized by heterotrophic bacteria during the sinking process (Boyd et al., 2004; Smetacek et al., 2012), and the phytoplankton biomass or derived detritus can become an important food source for heterotrophic bacteria in coastal dark waters and surface sediment (Billett et al., 1983; Martin et al., 2011; Braeckman et al., 2019).

Various bacterial populations of Flavobacteria, Roseobacter, and Gammaproteobacteria (e.g., *Alteromonas*) display successive relationships in the decomposition of phytoplankton-derived organic matter (Teeling et al., 2012; Zheng et al., 2018). The biological availability of phytoplankton-derived substrates shapes the heterotrophic bacterial community structure, lifestyle strategies, and responding periods (Teeling et al., 2012; Amin et al., 2015; Hahnke et al., 2015). Polysaccharides are produced by marine phytoplankton such as diatoms and haptophytes (Alderkamp et al., 2007), and these compounds constitute an important organic carbon source for heterotrophic bacterial communities (Muhlenbruch et al., 2018; Kappelmann et al., 2019). Flavobacteria, a taxon that produces diverse and large quantities of carbohydrate-active enzymes (CAZymes) and TonB-dependent transporters are specialized in the utilization of high molecular weight carbohydrate organic matter (Fernandez-Gomez et al., 2013).

The importance of marine bacterioplankton, including *Prochlorococcus* and *Synechococcus*, in oceanic primary production and carbon cycling has been well recognized in recent years (Flombaum et al., 2013; De Martini et al., 2018). In some coastal environments, *Synechococcus* accounts for 20% of the primary productivity (Li, 1994). Cyanobacteria (mainly *Synechococcus*) blooms are frequently observed in coastal waters and can drastically influence the cycling of carbon and other nutrients in eutrophic ecosystems (Glover et al., 1988; Suikkanen et al., 2010; Engstrom-Ost et al., 2015; Shi et al., 2017). Different cyanobacterial components (e.g., pigments, proteins, polysaccharides, and lipids) display different biological availabilities during decomposition (Fallon and Brock, 1979; Zhao et al., 2017; Zheng et al., 2019). However, the majority of *Synechococcus*-derived organic matter (SOM) is labile for heterotrophic bacteria, and N-, P-, and S-containing organic matter exhibit a much shorter turnover time (Shi et al., 2017; Zheng et al., 2019). How coastal microorganisms respond to the pulse increases of SOM during *Synechococcus* sp. bloom? How to link the microbial population succession and different biological available phytoplankton-derived organic matters?

Xiamen Island is a subtropical island, located on the southeastern coast of China. The abundance of *Synechococcus* may be comparable with that of total heterotrophic bacteria in the summer around Xiamen coastal areas, and these organisms are important contributors to primary production in this eutrophic system (Wang et al., 2019). Here, we conducted a SOM decomposition experiment with Xiamen coastal microbes to investigate (1) the processes underlying the degradation and changes in bioavailability of SOM throughout incubation, (2) the responses and behaviors of different microbial taxa and succession of the microbial communities over time, and (3) the coupling of biotic and abiotic processes occurred during incubations after addition of SOM. Our study advances our understanding of the turnover processes of phytoplankton-derived organic matter in coastal environments and provides clear evidence for microbially mediated biogeochemical cycles.

## Materials and Methods

### Experimental Setup and Sampling

**FIGURE 1:**
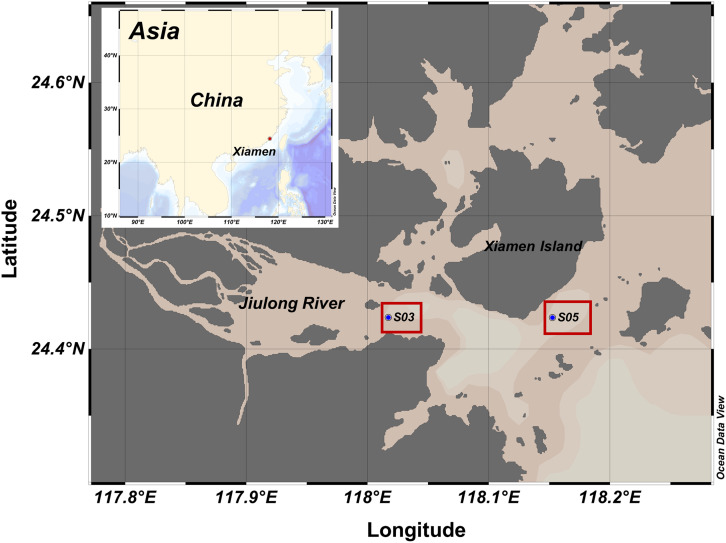
Sampling locations (station S03 and S05) around Xiamen Island, China.

*Synechococcus* sp. XM-24 was isolated from the coastal region near Xiamen Island (Zheng et al., 2018). *Synechococcus* sp. XM-24 (not axenic) was grown in the SN medium (Waterbury et al., 1986). Cells were collected via centrifugation at the exponential phase. The cells were then disrupted by freeze–thaw cycles to obtain *Synechococcu*s-derived organic matter (SOM). Seawater was collected from a depth of 5 m in the coastal region near Xiamen Island (stations S03 and S05) on June 29, 2017 (Figure 1). Geographically, station S03 is located in the mouth of the Jiulong River, which is a major source of freshwater to the Xiamen coastal area that is greatly impacted by terrestrial organic matter and inorganic nutrients. Station S05 is located south of Xiamen Island and is influenced by saline water from the South China Sea (Wang et al., 2019). The differing environmental characteristics and initial conditions are listed in the Supplementary Information (Supplementary Tables S1, S2). The experiment was carried out in 10 L-polycarbonate carboys that were pre-acid-washed. Each microcosm was established with 10 L seawater that was pre-filtered through a 3 μm filter (Millipore, Bedford, MA, United States) to remove eukaryotic cells. Considering the variable range of total organic carbon (TOC) concentrations in our studied region were from 80 to 200 μmol C L^–1^, as well as the background TOC of S03 and S05 with 90–100 μmol C L^–1^, we decided to add ∼60 μmol C L^–1^ SOM referred to our previous study (Zheng et al., 2019). The 60 μmol C L^–1^ organic carbon is approximately equal to the cellular carbon of ∼10^7^ *Synechococcus* sp. cells mL^–1^ based on 20 fg C cell^–1^ (Lee and Fuhrman, 1987; Benner, 1991), which is much higher than its peak number (∼10^5^ cells mL^–1^) in our studied regions (Wang et al., 2019). However, the highest abundance of marine picocyanobacteria ever reported were found in the Comacchio lagoon system (ranging from 1.2 to 2.4 × 10^7^ cells mL^–1^) (Sorokin and Zakuskina, 2010) and the Costa Rica dome (ranging from 1.2 to 3.7 × 10^6^ cells mL^–1^) (Saito et al., 2005; Ahlgren et al., 2014) suggesting their significance to coastal ecosystems. In this study, we amplified *Synechococcus* sp. contribution to local primary production in order to simulate microbial response to pulse increases of SOM from possible *Synechococcus* spp. blooms occurred in the coastal regions. For each set of our incubations, ∼60 μmol L^–1^ SOM was added to three carboys, which served as the SOM-addition group, and two carboys without added SOM served as a control group. Microcosms were incubated at 28 ± 0.5°C in the dark.

Incubations were sampled at days 0, 1, 2, 4, 7, 20, 50, 80, and 180. For TOC samples, 20 mL samples were collected directly into 40 mL glass vials (CNW, Germany) and immediately stored at −20°C for further analysis. For dissolved organic carbon (DOC) and fluorescent dissolved organic matter (FDOM) samples, 20 mL samples were filtered through pre-combusted (450°C, 4 h) 0.7 μm pore-size GF/F filters (47 mm diameter, Whatman, Maidstone, United Kingdom) into 40 mL glass vials and stored at −20°C. To prevent carbon contamination, all glass materials used for sample collection and storage were acid-washed, Milli-Q (ion- and nuclease-free water) rinsed, and pre-combusted for 4 h at 450°C. For inorganic nutrients measurement, 50 mL samples were filtered through 0.45 μm polycarbonate membrane filters (47 mm diameter, Millipore, United States) and stored at −20°C for further analysis. For DNA and RNA samples, 300 mL water samples were immediately filtered through 0.2 μm polycarbonate membrane filters (47 mm diameter, Millipore, United States). For the control incubations, 300 mL of water samples were immediately filtered through 0.2 μm polycarbonate membrane filters (47 mm diameter, Millipore, United States) for DNA and RNA analyses. However, for the SOM-addition incubations 300 mL of water samples was filtered through 3 and 0.2 μm polycarbonate membrane filters to obtain two size fractions, the >3 μm (particle attached bacteria) and the 0.22–3 μm (free-living bacteria). This fractionation was performed because in the *Microcystis aeruginosa* bloom, it has been observed that bacteria attached to cyanobacterial cells or detritus during the decline phase of the bloom (Liu et al., 2019). This fractionation was not applied in the control incubations because the seawater was pre-filtered through a 3 μm filter before the incubations, and no SOM was added. Samples for RNA extraction were collected within 30 min and stored in 2-mL RNase-free tubes with RNA stabilization solution (Ambion, United States). All filters were flash-frozen in liquid nitrogen for 10 min and subsequently stored at −80°C until DNA or RNA extraction.

### Analysis of TOC, DOC, and Dissolved Inorganic Nutrient Concentrations

Total organic carbon and DOC concentration were measured by high temperature catalytic oxidation (HTCO) using a Shimadzu TOC-VCPH analyzer (Japan). Concentration of dissolved inorganic nutrients, including nitrite (NO_2_^–^), nitrate (NO_3_^–^), and phosphate (PO_4_^3–^), were measured via spectrophotometric methods (Knap et al., 1996) using a Technicon AA3 Auto Analyzer (Bran+Luebbe, GmbH, Germany). Indophenol blue (IPB) spectrophotometric methods were used to analyze ammonium (NH_4_^+^) concentration in samples (Ma et al., 2014). Although DOC samples were taken in phases II and III, they were contaminated and thus not shown.

### Excitation Emission Matrix Fluorescence

Fluorescence measurements were performed using a 1 cm quartz cuvette and a Varian Cary Eclipse spectro-fluorometer (United States). Emission spectra were scanned every 2 nm at wavelengths from 280 to 600 nm, with excitation wavelengths ranging from 240 to 450 nm at 5 nm intervals (Wang et al., 2017). Slit widths were 10 nm for both excitation (ex) and emission (em). The EEM of Milli-Q water scanned on the same day was subtracted from the samples’ EEMs. The fluorescence intensities were corrected to the area under the water Raman peak of Milli-Q water (excitation = 350 nm) and calibrated to Raman Unit (RU) (Lawaetz and Stedmon, 2009). EEMs were decomposed into components using parallel factor analysis (PARAFAC), with MATLAB 2012 and the DOMFluor toolbox (Stedmon et al., 2003; Stedmon and Bro, 2008).

### DNA and RNA Extraction, PCR, and Sequence Processing

DNA extraction was performed using the phenol-chloroform-isoamyl alcohol method (Massana et al., 2000). The quantity and quality of extracted DNA were measured using a NanoDrop ND-1000 spectrophotometer (Thermo Fisher Scientific, Waltham, MA, United States) and agarose gel electrophoresis, respectively. RNA was extracted using TRIzol reagent (Invitrogen, United States). RNA quality was verified using agarose gel electrophoresis. First-strand cDNA was generated using a SuperScript First-Strand Synthesis System (Invitrogen, United States) with random primers, followed by synthesis of the second-strand cDNA using RNase H and DNA polymerase I. DNA and cDNA amplification of the bacterial 16S rRNA genes (V4–V5 region) was performed using the forward primer 515F (5′-GTGCCAGCMGCCGCGGTAA-3′) and the reverse primer 907R (5′-CCGTCAATTCMTTTRAGTTT-3′). Thermal-cycling began with an initial denaturation at 98°C for 1 min, followed by 30 cycles of denaturation at 98°C for 10 s, annealing at 50°C for 30 s, and elongation at 72°C for 60 s, with a final extension at 72°C for 5 min. Sequencing libraries were generated using an NEB Next Ultra DNA Library Prep Kit (NEB, United States) for Illumina following the manufacturer’s recommendations, and index codes were added. Library quality was assessed on a Qubit^@^ 2.0 Fluorometer (Thermo Scientific, United States) and Agilent Bioanalyzer 2100 system (United States). Finally, the library was sequenced on an Illumina HiSeq 2500 platform, generating 450 bp paired-end reads that were then combined using FLASH software (V1.2.7^1^). Raw data were first quality-filtered with QIIME to remove reads that did not meet the desired quality (Caporaso et al., 2010). Chimeras were removed using the Chimera Slayer algorithm in MOTHUR (Haas et al., 2011). Operational taxonomic units (OTUs) were clustered with a 97% similarity cutoff using UPARSE software (UPARSE, v7.0.1001^2^). The OTUs were taxonomically classified based on the SILVA database (Version 132). Sequence data were deposited in the National Center for Biotechnology Information (NCBI) Sequence Read Archive^3^ under BioProject PRJNA532855.

### Statistical Analysis

Non-metric multidimensional scaling (NMDS) was used to determine the similarity of samples to each other based on Bray-Curtis similarities (Clarke and Warwick, 1994) with CANOCO software (Version 5.0) (Terbraak, 1989). Bray-Curtis similarities were calculated based on relative abundance matrices of OTUs for communities. Dimension reduction was achieved by taking the original set of samples and calculating Bray–Curtis similarity (distance) for each pairwise comparison. The samples were then represented graphically in two dimensions such that the distance between points on the plot approximates their multivariate similarity as closely as possible.

## Results

### Variations of Organic Carbon Concentration During Incubations

**FIGURE 2:**
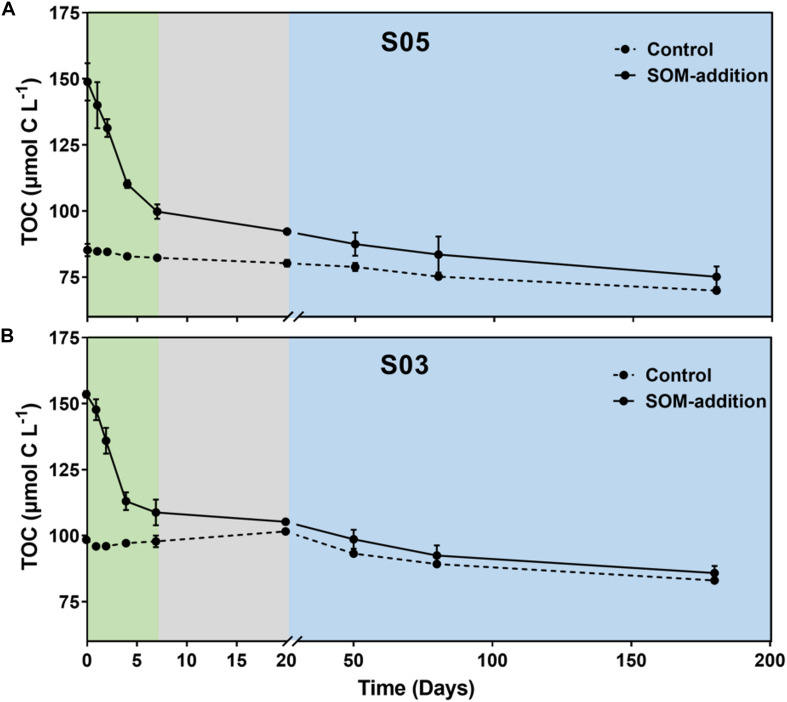
Variation in TOC concentrations throughout incubations at stations **(A)** S05 and **(B)** S03. Three phases (I, II, and III) were identified based on the rate of TOC consumption in the SOM-addition groups. Phase I: from 0 to 7 days, indicated by the green shading; Phase II: from 8 to 20 days, indicated by the gray shading; and Phase III: from 21 to 180 days, indicated by the blue shading. Error bars represent the range of values from triplicate measurements for SOM-addition groups and duplicate measurements for the control groups.

*Synechococcus*-derived organic matter was quickly consumed by bacteria during the 180-day incubations, and TOC concentration continually decreased from 148.9–153.5 to 75.2–85.9 μmol C L^–1^ in the SOM-addition groups and from 85.2–98.4 to 69.9–83.1 μmol C L^–1^ in the control groups (Figure 2). According to the TOC consumption rate in the SOM-addition groups, the 180-day incubation period could be partitioned into three phases: phase I (0–7 days), phase II (8–20 days), and phase III (21–180 days). Considering the similar variation patterns in terms of TOC concentration, inorganic nutrient re-generation, and total/active microbial community in the two-station incubation systems, we mainly focused on the station S05 incubation systems for the following discussions.

In the station S05 incubation system (Figure 2A), the TOC concentration decreased by 49.0 ± 4.8 μmol C L^–1^ (from 148.9 ± 5.8 to 99.8 ± 2.3 μmol C L^–1^) with a consumption rate of 7.01 ± 0.29 μmol C L^–1^ day^–1^ in phase I. The TOC concentration ranged from 99.8 ± 2.3 to 92.3 ± 0.8 μmol C L^–1^ in phase II with a consumption rate of 0.53 ± 0.10 μmol C L^–1^ day^–1^. The TOC concentration reduced to 75.2 ± 3.2 μmol C L^–1^ at the end of the incubations with a consumption rate of 0.10 ± 0.01 μmol C L^–1^ day^–1^. Compared to the SOM-addition groups, the TOC concentration had a relatively narrow range variation from 85.2 ± 1.6 to 69.9 ± 0.5 μmol C L^–1^ in the control groups during the incubations.

In the S03 incubations (Figure 2B), the TOC concentration decreased from 153.5 ± 1.0 to 108.8 ± 4.0 μmol L^–1^ with a consumption rate of 6.38 ± 0.54 μmol C L^–1^ day^–1^ in phase I. In phase II, the TOC concentration decreased to 105.5 μmol L^–1^ with a consumption rate of 0.64 μmol C L^–1^ day^–1^. The TOC concentration dropped to 85.9 ± 2.2 μmol C L^–1^ at day 180 with a consumption rate of 0.13 ± 0.03 μmol C L^–1^ day^–1^ in phase III. The TOC concentration decreased by 15.4 ± 0.3 μmol C L^–1^ from 98.4 ± 0.7 to 83.1 ± 0.3 μmol C L^–1^ in the control groups over the course of the incubations.

DOC concentrations were also monitored in our incubaitons (Supplementary Figure S1). Although the TOC concentrations dramatically decreased during the first 7 days, DOC concentrations exhibited few variations in the SOM-addition groups, suggesting particulate organic carbon (POC) was a major component of SOM that was rapidly consumed by microbes in phase I. The TOC primarily comprised DOC over the incubations in controls and after day 7 in treatments.

### Variations of Inorganic Nutrients During Incubations

Along with the consumption of SOM/TOC, variations of inorganic nutrients, including NH_4_^+^, NO_2_^–^, NO_3_^–^, and PO_4_^3–^, were measured during the 180-day incubations (Supplementary Figures S2, S3).

In the S05 incubations, the NH_4_^+^ concentration gradually increased from 4.3 ± 0.2 to 14.0 ± 0.3 μmol L^–1^ in phase I, then continually reduced to 1.8 ± 0.4 μmol L^–1^ at the end of phase II and remained low (∼1.0 μmol L^–1^) in phase III in the SOM-addition groups (Supplementary Figure S2A). The concentration of NO_2_^–^ was low (∼2.0 μmol L^–1^) in phase I, then increased to 17.7 ± 1.9 μmol L^–1^ at the 20th day and decreased back to a very low level (undetectable) from day 50. The NO_3_^–^ concentration showed few variations before phase III ranging from 31.9 ± 0.4 to 28.4 ± 3.6 μmol L^–1^ (the 20th day), then gradually increased from 28.4 ± 3.6 to 52.7 ± 2.6 μmol L^–1^ during phase III (especially from days 20 to 50), and then remained with relatively small variations in the following days. Clear transformation processes of nitrogen-containing nutrients from SOM (N-containing organic matter) to NH_4_^+^ in phase I, from NH_4_^+^ to NO_2_^–^ in phase II, and from NO_2_^–^ to NO_3_^–^ in phase III were observed. In the control groups, the concentration of NH_4_^+^ (from 4.7 ± 0.5 to 0.8 ± 0.1 μmol L^–1^) and NO_2_^–^ (from 2.0 ± 0.1 μmol L^–1^ to undetectable at day 180) gradually decreased over the course of the entire incubations. Meanwhile, the NO_3_^–^ concentration gradually increased from 26.1 ± 0.09 to 33.2 ± 0.1 μmol L^–1^ at the end of the incubations. The PO_4_^3–^ concentration increased from 1.0 ± 0.04 to 1.5 ± 0.02 μmol L^–1^ in phase I (Supplementary Figure S3A). Only small fluctuations in PO_4_^3–^ concentration, ranging from 1.4 ± 0.1 to 1.9 ± 0.01 μmol L^–1^, were detected in phases II and III. Compared to SOM-addition groups, the concentration of PO_4_^3–^ had a relatively narrow range of variation, from 0.6 ± 0.02 to 0.8 ± 0.004 μmol L^–1^, in the control groups during the incubations.

The incubations from station S03 seawater displayed a similar inorganic nutrients variation pattern with that in station S05 (Supplementary Figures S2B, S3B). In the SOM-addition groups, NH_4_^+^ concentration increased from 8.1 ± 0.5 to 16.8 ± 0.5 μmol L^–1^ in phase I and decreased to 2.4 ± 1.1 μmol L^–1^ at the 20th day (Supplementary Figure S2B). The concentration of NO_2_^–^ increased to a peak value (17.1 ± 5.0 μmol L^–1^) at the 20th day before being reduced to a low level (0.2 ± 0.04 μmol L^–1^) at day 50. Additionally, the NO_3_^–^ concentration increased from 53.3 ± 0.9 (on the initial day) to 67.6 ± 7.9 μmol L^–1^ (at the 50th day) and reached 81.5 ± 0.4 μmol L^–1^ by the end of the incubations. The concentration of PO_4_^3–^ increased from 1.3 ± 0.06 to 1.7 ± 0.07 μmol L^–1^ in phase I and reached 1.8 ± 0.1 μmol L^–1^ by the end of the incubations (Supplementary Figure S3B). In the control groups, the concentration of NH_4_^+^ and NO_2_^–^ gradually decreased, from 8.4 ± 0.4 to 1.0 ± 0.1 μmol L^–1^ and from 3.4 ± 0.01 μmol L^–1^ to undetectable levels, respectively. The concentration of NO_3_^–^ exhibited relatively small variations during the entire incubation. The PO_4_^3–^ concentration increased from 1.0 ± 0.03 to 1.3 ± 0.02 μmol L^–1^ over the course of the entire incubations.

### Variations of FDOM Components During Incubations

**FIGURE 3:**
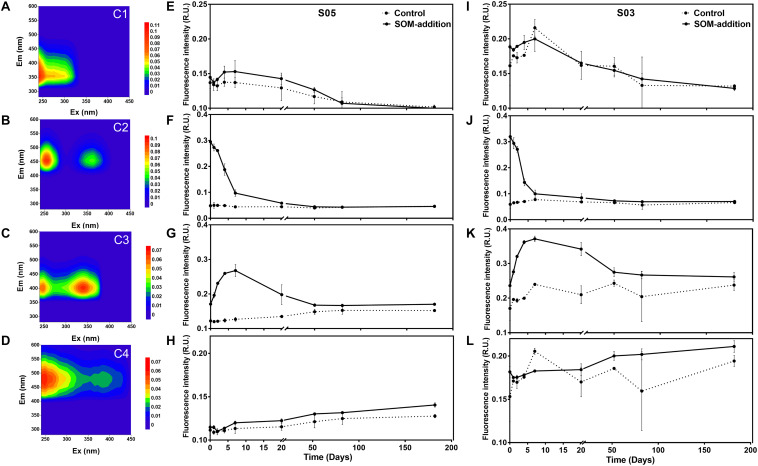
Characterization of FDOM components identified throughout the incubation experiments. Excitation emission matrices are shown for the C1, C2, C3, and C4 components on the left panels **(A–D)**, respectively. Variation in abundance patterns for each component for the four components of station S05 are in the middle panels **(E–H)**. Finally, variation in abundance patterns for each component of station S03 is shown in the right panels **(I–L)**.

Four distinct fluorescent components (C1, C2, C3, and C4) were identified by PARAFAC analyses in our incubations. Component C1 exhibited fluorescence properties similar to those of the tryptophan-containing molecules (also called protein-like compounds), whereas components C2, C3, and C4 showed locations of maximum peak intensities typical of what are referred to as humic-like molecules (Figure 3). Variation patterns for each component were the same between the two stations.

C1 was characterized as a protein-like component, displaying emission spectra with maxima below 400 nm, which had an excitation/emission maximum at 240/352 nm, representing the tryptophan-like fluorophore, which was initially categorized as T peak (Coble, 1996). The fluorescence intensity of C1 exhibited a slight fluctuation in phase I and then decreased until the end of the incubations (Figures 3E,I). C2 had an excitation/emission maximum at 255, 365/456 nm, and a similar fluorescent signal was also detected in a 15-day culture of *Synechococcus* sp. CB0101 (Zhao et al., 2017). The fluorescence intensity of C2 was low in the control groups and relatively stable over the course of the entire incubations (Figures 3F,J). In the SOM-addition groups, the fluorescence intensity of C2 dramatically decreased in phase I and continually decreased during phase II and until day 50. From this point (the 50th day) to the end of incubations, the fluorescence intensity of C2 was relatively stable. C3 displayed two excitation maxima at 250 and 340 nm and one emission maxima at 404 nm, which was initially categorized as the M humic-like peak (Coble, 1996). The fluorescence intensity of C3 increased in phase I reaching the highest value at day 7 before gradually decreasing during phase II and the early stage of phase III (before the 50th day), whereas it remained constant after day 50 (Figures 3G,K). C4 also displayed two excitation maxima at 250 and 385 nm and one emission maxima at 484 nm, which was initially categorized as the C humic-like peak (Coble, 1996). The humic-like C4 fluorescence intensity gradually increased over the entire incubation period (Figures 3H,L).

### Variations and Shifts of Microbial Communities

To explore the active microbial community responses and secession during incubations, a total of 140,041 and 138,633 sequences (16S rRNA) were obtained (after removing Cyanobacteria reads mainly from the SOM addition) on both size fractions from stations S05 and S03 incubations, respectively. Over the entire incubations, Gammaproteobacteria (28.83 and 26.72%, in station S05 and S03 incubations, respectively; the same below), Alphaproteobacteria (14.14 and 12.04%), Bacteroidetes (13.35% and 20.97%), Planctomycetes (9.29 and 7.80%), Thaumarchaeota (7.00 and 9.09%), and Actinobacteria (7.25 and 4.83%) were the dominant active microbial communities.

**FIGURE 4:**
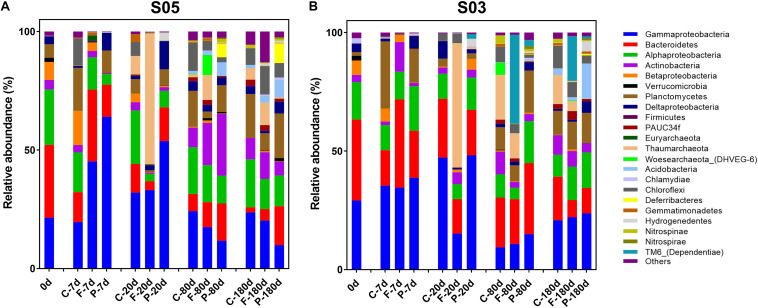
Active microbial community composition based on 16S rRNA variations at station **(A)** S05 and **(B)** S03 throughout the incubations, shown at the phylum level (except for Proteobacteria, which are divided into classes). C-, control; F-, 0.22–3 μm size fraction (free-living fraction); and P-, >3 μm size fraction (particle-associated fraction).

In the station S05 incubations (Figure 4A), the SOM was quickly consumed in phase I by microbes: Alphaproteobacteria (11.67 and 18.75%, on the 0.22–3 μm and the >3 μm size fractions, respectively; the same below), Gammaproteobacteria (45.18 and 64.04%), Bacteroidetes (30.35 and 13.52%), and Actinobacteria (2.72 and 0.34%) were dominant at day 7. Planctomycetes had a significantly higher relative abundance on the >3 μm size fraction (9.49%) than on the 0.22–3 μm size fraction (0.25%) in this phase. In phase II, Alphaproteobacteria, Gammaproteobacteria, Bacteroidetes, and Actinobacteria still accounted for 40.43 and 76.50% of the active microbes on the 0.22–3 and >3 μm size fractions, respectively. The relative abundance of Thaumarchaeota reached 55.25% on the 0.22–3 μm size fraction at day 20 and only was 0.17% on the >3 μm size fraction. In phase III, Alphaproteobacteria, Gammaproteobacteria, Bacteroidetes, and Actinobacteria accounted for 61.46 and 65.21% at day 80 of active microbes on the 0.22–3 and >3 μm size fractions, respectively, and 49.03 and 45.11% at day 180, respectively. Actinobacteria had a high relative abundance at day 80 of the active microbial community, with relative abundances of 17.82 and 26.01% on the 0.22–3 and >3 μm size fractions, respectively. Additionally, the relative abundance of Thaumarchaeota remained at 10.54 and 9.46% on the 0.22–3 μm size fraction at days 80 and 180, respectively. The relative abundance of Chloroflexi reached up to 12.02% at day 180 on the 0.22–3 μm fraction. Compared to the first two phases, the relative abundance of active Acidobacteria (2.85 and 7.26%, on the 0.22–3 and >3 μm size fractions, respectively; the same below) and Nitrospinae/Nitrospirae (0.40 and 1.46%) increased at day 180.

In the station S03 incubations, the active microbial responses and succession exhibited the similar pattern with that of in station S05 incubations (Figure 4B). Alphaproteobacteria (11.67 and 18.75%, on the 0.22–3 and >3 μm size fractions, respectively; the same below), Gammaproteobacteria (34.54 and 38.73%), and Bacteroidetes (37.29 and 19.87%) were dominant the active microbes in phase I on both size fractions. Moreover, Actinobacteria also dominated at day 7, with a relative abundance of 12.47% on the free-living size fraction, while Planctomycetes became abundant on the >3 μm size fraction and accounted for 14.26% of active microbes. At day 20, Thaumarchaeota became the most dominant active microbial group (52.48%) on the 0.22–3 μm size fraction. The TM6-Dependentiae group displayed relatively high activity on the 0.22–3 μm size fraction in phase III with relative abundances of 37.63 and 18.85% at days 80 and 180, respectively. Acidobacteria represented 14.89% of active microbes at day 180 on the >3 μm size fraction, while its relative abundance was <1% in the first two phases. Additionally, Chloroflexi (6.41 and 3.38%, on the 0.22–3 and >3 μm size fractions, respectively; the same below), Hydrogenedentes (0.12 and 4.34%), Gemmatimonadetes (0.05 and 1.02%), and Nitrospinae/Nitrospirae (0.27 and 1.18%) exhibited relatively high activities at day 180 compared to the first two phases.

The variations of total microbial communities were also studied, and a total of 195,542 and 200,153 sequences (16S rDNA) were also obtained (after removing Cyanobacteria reads mainly from the SOM addition) from stations S05 and S03 incubations, respectively. Considering the similar microbial response pattern with active microbial populations, these results were put into Supplementary Material and Supplementary Figure S4.

**FIGURE 5:**
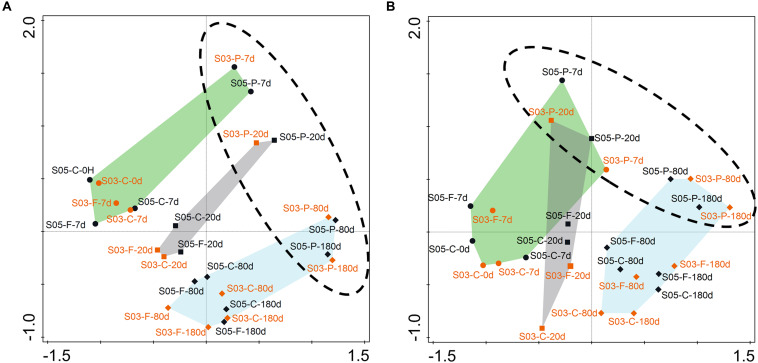
Non-metric multidimensional scaling (NMDS) ordination based on Bray–Curtis similarities between microbial communities at the two-station incubations. **(A)** rDNA- based; **(B)** rRNA-based. Each dot represents an individual sample in the NMDS figures, S05: dark dot; S03: orange dot. C-, control; F-, 0.22–3 μm size fraction (free-living fraction); and P-, >3 μm size fraction (particle-associated fraction). Phase I: indicated by the green shading; Phase II: indicated by the gray shading; and Phase III: indicated by the blue shading. The phase I contained samples from days 0 and 7; the phase II only comprised the 20th samples; and the phase III consisted of samples from days 80 and 180.

The NMDS analyses showed that total (Figure 5A) and active (Figure 5B) microbial community composition from the two–station incubations could be divided into three clusters, corresponding to the three phases based on organic matter consumption rates. Meanwhile, the microbial responses and community composition displayed high similarity at the same time point and size fraction between the two-station incubations in SOM-addition groups. Moreover, the relative contribution of specific OTUs to the Bray–Curtis index of dissimilarity was calculated using SIMPER (Easson and Thacker, 2014). The results showed Alphaproteobacteria, Gammaproteobacteria, Flavobacteriia, Acidimicrobiia, and Planctomycetacia were primarily responsible for the period division during the incubations at both the 16S rDNA and 16S rRNA levels (Supplementary Table S3). Furthermore, the free-living microbial communities (on the 0.22–3 μm size fraction) were clustered together, with no size-fraction samples in the control groups. The particulate-attached microbial communities (on the >3 μm size fraction) were separated from them, reflecting their different trophic strategies in the incubations.

## Discussion

### Quantity and Quality Changes of Organic Matter in Incubations

Marine phytoplankton release large amounts of photosynthetic products directly into the surrounding water via passive and active exudation. These processes are responsible for the accumulation of vast amounts of microbial biomass (Muhlenbruch et al., 2018). Phytoplankton biomass is an important source of particulate organic matter in the ocean and also contributes to the POM sinking with the biological pump (Herndl and Reinthaler, 2013). In our incubation experiments, approximately 60 μmol C L^–1^ SOM, mainly in the form of POM, was added to the SOM-addition groups. Three phases could be clearly separated based on the organic carbon consumption rates in the SOM-addition groups. SOM was rapidly degraded by coastal heterotrophic microbes in phase I and II, suggesting its labile properties. Meanwhile, the concentration of NH_4_^+^ and PO_4_^3–^ rapidly accumulated with the consumption of SOM in phases I and II, and showed a significant negative correlation with TOC concentration, respectively (Supplementary Figure S5). This indicated that SOM was enriched with nitrogen (N)-containing and phosphorus (P)-containing organic matter (Fiore et al., 2015; Tada and Suzuki, 2016; Ma et al., 2018). Organic matter containing N and P (e.g., amino acids and oligopeptides) are usually more labile for bacterial metabolism (Rossel et al., 2015; Zhao et al., 2017; Zheng et al., 2019).

The intensity variations of four FDOM components indicated the property (i.e., bioavailability) changes of DOC over the 180-day incubations. C1 presented a protein-like component, and its intensity variations displayed a close relationship with heterotrophic bacterial growth activities. It seems that component C1 was produced/released during high bacterial metabolic activities and was re-used after the environmental labile organic matter was depleted. Previous studies have also reported marine bacteria can mediate the production and consumption of peak T, and the fluorescence of peak T and the bacterial biomass had a significant positive linear correlation (Romera-Castillo et al., 2011).

The maximum intensity of humic-like C2 components was found at the initial point in the SOM-addition groups, which indicates its origin was from disrupted *Synechococcus* sp. cells. The same fluorescent signal was also observed in a 15-day culture of *Synechococcus* sp. CB0101 (Zhao et al., 2017). The intensity of C2 rapidly decreased in phase I, corresponding to a quick consumption of TOC/SOM, suggesting its labile properties with a rapid turnover rate.

Humic-like C3 and C4 components have been widely observed in estuary and oceanic samples (Stedmon and Markager, 2005; Yamashita et al., 2010; Jorgensen et al., 2011; Wang et al., 2017). In the open-ocean water column, the vertical distribution of these two humic-like fractions typically exhibited the lowest fluorescence at the surface and increased with depth to a relatively constant level in the intermediate and deep waters (Jorgensen et al., 2011; Wang et al., 2017). The low fluorescence intensities observed in the surface layer were likely due to photochemical degradation, given that humic-like compounds are sensitive to UV light (Mopper et al., 1991; Nieto-Cid et al., 2006). Our incubations were carried out under completely dark conditions, and the continuously accumulated C3 intensity was observed in phase I, corresponding to a quick consumption of SOM/TOC in this period. It decreased from day 7 to the end of the incubations. In previous studies, C3 has been reported to be produced by phytoplankton isolates (Romera-Castillo et al., 2010) or by a single bacterial strain (Goto et al., 2017) and could also be partially consumed by environmental microbes (Romera-Castillo et al., 2011). Our results suggest that humic-like C3 can be produced by some bacterial groups during quick utilization of SOM and then re-used by other bacterial groups after the labile organic matter was depleted. This indicates the bio-availability of component C3 was dependent on the available organic carbon in the incubations. Thus, component C3 could be categorized as semi-labile dissolved organic matter (SLDOM) compounds. Component C4 exhibited a slow but continuous accumulation without obvious phase separation, and no signs of re-utilization were observed, indicating its biological recalcitrant characteristics, which could be categorized as refractory dissolved organic matter (RDOM) molecules. Previous results have also observed that peak C could be continuously produced by marine bacteria in a 30-day incubation with phytoplankton exudates (Romera-Castillo et al., 2011).

### Microbial Responses/Behaviors of Different Taxa During Incubations

Generally, the addition of SOM did not trigger dramatic changes in the microbial community composition between the control and SOM-addition groups. These results were expected, given that *Synechococcus* was one of the main contributors to primary production in summer around Xiamen coastal areas (Wang et al., 2019) and that *Synechococcus* sp. XM-24 was isolated from this region (Zheng et al., 2018). Based on NMDS analyses, the particulate-attached microbial communities were separated from free-living microbial communities and no size-fraction samples in the control groups. These results were consistent with previous studies that demonstrated that the majority of bacteria in the environment were free-living and that particle-attached bacteria generally accounted for less than 20% of total bacteria during phytoplankton blooms, and even less in natural seawaters (Azam et al., 1983; Simon et al., 2002; Ghiglione et al., 2007). Although the total microbial communities on the free-living size fraction were clustered together with microbes in the control groups, the active microbial communities on the 0.22–3 μm size fraction were relatively independent at both stations (Supplementary Figure S6). This suggests that the pulse addition of SOM markedly changed microbial growth and metabolic activities. Variations of active microbial communities could reflect environmental microbial metabolic conditions on a fine scale.

Generally, Alphaproteobacteria, Gammaproteobacteria, and Bacteroidetes dominated the communities throughout the incubations, especially in phase I, suggesting that these taxa were the major participators in the degradation of SOM. Members of Alphaproteobacteria, primarily *Roseobacter* and SAR11 clades in the ocean, can function in diverse and flexible metabolic roles (Zheng et al., 2018) and are specialized in processing low-molecular-weight dissolved organic substrates (Moran et al., 2003; Alonso and Pernthaler, 2006). Gammaproteobacteria is often defined as an opportunistic group, with a broad range of potential substrates that can show clear responses during phytoplankton blooms (Tada et al., 2011; Sarmento and Gasol, 2012). Bacteroidetes has been widely reported as being specialized in degrading high-molecular weight dissolved and particulate organic substrates and commonly detected as a dominant group during phytoplankton blooms (Cottrell and Kirchman, 2000; Teira et al., 2008; Teeling et al., 2012; Zheng et al., 2018). These three bacterial groups are ubiquitous and abundant phylogenetic clades in global oceans, and they harbor their own unique metabolic strategies to adapt to the environment. Moreover, during the long-term incubation, there was also a marked succession of phylotypes within these groups (Supplementary Figures S7–S9).

Abundant Thaumarchaeota were observed from day 20, both in the 16S rDNA and rRNA level, suggesting high activity in SOM-addition groups. The relative abundances accounted for more than half of active microbes at day 20 in the free-living size fraction and then maintained ∼20% relative abundances of total and active microbes in phase III. Thaumarchaeota are one of the most abundant microbial cells in the ocean, especially in the aphotic zone (Karner et al., 2001; Teira et al., 2006; Santoro et al., 2015). The success of Thaumarchaeota in phases II and III likely was due to their ability to fix inorganic carbon (Herndl et al., 2005). The labile organic carbon-limited environment and high concentration of NH_4_^+^, which came from the consumption of SOM, provide a chance for revival for Thaumarchaeota in phase II. As the transformation process from NO_2_^–^ to NO_3_^–^ was observed in our incubations, the nitrite-oxidizing bacteria Nitrospinae/Nitrospirae were detected. Nitrospinae/Nitrospirae are important for chemoautotrophy in the dark ocean, and these organisms are the most abundant and globally distributed nitrite-oxidizing bacteria in the ocean (Pachiadaki et al., 2017). Although the concentration of NH_4_^+^ and NO_2_^–^ became extremely low after day 20, it seems three forms of inorganic nitrogen nutrients reached a dynamic balance in the incubations. This suggests that an ammonia-oxidizing process was still occurring. Cultivated Thaumarchaeota strains have been reported to be adapted to oligotrophic conditions with low ammonium concentration (in the nM to μM range) (Martens-Habbena et al., 2009; Prosser and Nicol, 2012; Bayer et al., 2016). Here, Thaumarchaeota were mostly found on the 0.22–3 μm size fraction, while Nitrospinae/Nitrospirae were mainly detected on the >3 μm size fraction in our incubations. The reciprocal feeding model between marine nitrite-oxidizing bacteria and ammonium-oxidizing archaea was proposed by Pachiadaki et al. (2017). However, the concentration of urea and cyanate were not measured in this study. Previous reports have shown that the biovolume of marine Nitrospinae is 50 times larger than that of Thaumarchaeota (Spieck et al., 2014; Pachiadaki et al., 2017). Thaumarchaeota could function via chemoautotrophic growth on ammonia, and an obligate mixotrophy lifestyle was discovered for *Nitrosopumilus* sp. strains (Qin et al., 2014).

In the later period of our incubations, some species that were rare in the early phases became abundant, including Acidobacteria, Chloroflexi, Gemmatimonadetes, and Nitrospinae/Nitrospirae. These specific taxa have been commonly detected as dominant populations in the deep sea and in surface sediment (Morris et al., 2004; Quaiser et al., 2008; Kouridaki et al., 2010; Schauer et al., 2010). Acidobacteria are highly diverse and ubiquitous in the ocean, especially abundant in the deep sea, and have been shown to be the most prevalent bacterial groups in the deep-sea surface sediments (Quaiser et al., 2008; Kouridaki et al., 2010). The limited isolates indicate that Acidobacteria have relatively large genome sizes, up to 10 Mbp, and they encode a series of CAZymes involved in the decomposition of various biopolymers (Quaiser et al., 2008). The Chloroflexi SAR406 and SAR202 clusters, previously described as ubiquitous in meso- and bathypelagic seawaters, are another abundant group in the dark ocean (Morris et al., 2004). Cultures in the phylum Chloroflexi showed diverse phenotypes, including anoxygenic phototrophs (green non-sulfur bacteria), aerobic thermophiles, and anaerobic halorespirers (Varela et al., 2008; Galand et al., 2010). Moreover, Chloroflexi and Gemmatimonadetes were also reported as dominant microbial groups in the sediments of the Mariana Trench (Nunoura et al., 2018). However, some of these taxa (e.g., Acidobacteria and Gemmatimonadetes) are also major phyla in terrestrial environments (DeBruyn et al., 2011). Here, the estuarine and coastal ecosystem was influenced by an inflow of freshwater as well as an input of terrestrial bacteria resulting in the inability of avoiding a few terrestrial populations from growing.

### Coupling Between C and N Metabolic Processes Mediated by Coastal Microbes

**FIGURE 6:**
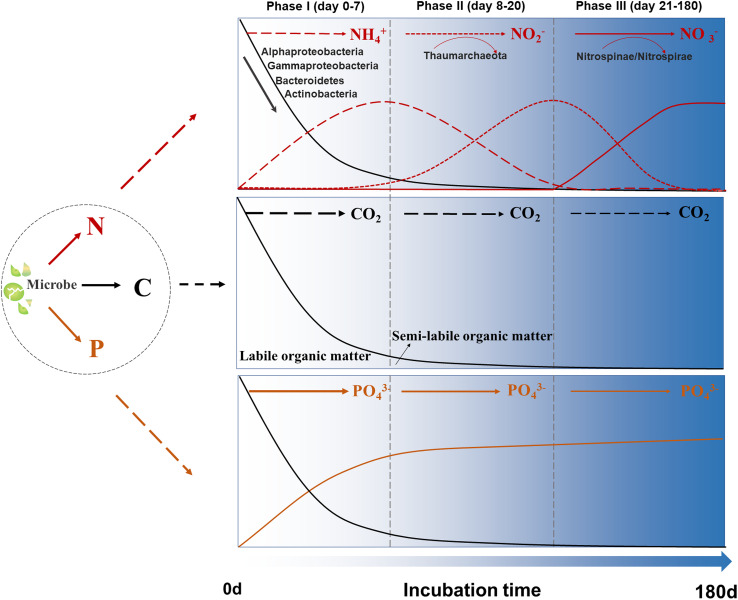
Schematic illustration of C, N, and P coupling in our 180-day incubation system. Black lines: the variations of total organic carbon concentrations; Red lines: the transformation of three forms of inorganic N; Orange lines: the variations of phosphate concentrations.

Our study clearly illuminated the coupling between C and N metabolic processes mediated by coastal microbes during the incubations (Figure 6). In phase I, ∼80% SOM was quickly utilized by microbes corresponding to the LDOM component C2 rapidly decreasing. Meanwhile, the SLDOM component C3 greatly accumulated in this phase. It is reported that viral lysates from infected *Synechococcus* sp. cells could be a significant source of high molecular weight dissolved organic nitrogen compounds, and abundant peptides from proteolysis of the light-harvesting protein phycoerythrin were detected (Ma et al., 2018). In addition, both the Fourier transform ion cyclotron resonance mass spectrometry and Nuclear magnetic resonance spectroscopy revealed N-containing compounds were abundantly present in *Synechococcus* sp. DOM (Zhao et al., 2017). These labile organic matters comprising abundant N-containing molecules in the SOM fueled the heterotrophic bacterial metabolic activity in the early periods. Alphaproteobacteria, Gammaproteobacteria, and Bacteroidetes, as well as endemic abundant bacterial groups (e.g., Actinobacteria) were the major contributors in phase I. As the concentration of NH_4_^+^ and PO_4_^3–^ rapidly accumulated in this phase, the microbial metabolic activities drove the re-cycle of N and P, from the organic form to inorganic form. NH_4_^+^ reached a maximum value at day 7 in all SOM-addition groups, while the NO_2_^–^ and NO_3_^–^ did not display clear variations during phase I, suggesting that N-containing SOM was directly regenerated as NH_4_^+^ in the incubations.

In phase II, the organic matter consumption rate became much lower, and ∼12% of SOM was degraded. LDOM component C2 slowly reduced, and SLDOM component C3 was re-utilized. After most labile organic matters were depleted, as well as the accumulation of NH_4_^+^ in phase I, Thaumarchaeota became a dominant group and drove transformation process of N from NH_4_^+^ into NO_2_^–^ in phases II. Meanwhile, the highest Thaumarchaeota activities corresponded the highest NO_2_^–^ concentration at day 20 in the SOM-addition groups. The incubation systems displayed a transformation from heterotrophy to mixotrophy lifestyles under dark incubations which was similar with the vertical distribution of heterotrophic bacteria/chemoautotrophic archaea from eutrophic to dark ocean ecosystem (Church et al., 2003; De Corte et al., 2009).

In phase III, after most biological available organic matters was utilized, the TOC consumption rate was even lower. The transformation process from NO_2_^–^ to NO_3_^–^ was observed. Nitrospinae/Nitrospirae, as well as some other unknown microbial groups might contribute to this process, continually transformed NO_2_^–^ into NO_3_^–^ and obtained energy from the nitrite-oxidizing processes. Although bacteria mediated heterotrophic C, N, and P cycling was commonly found during microbial utilization of phytoplankton derived organic matters (Haaber and Middelboe, 2009), the entire incubation period in this study showed a perfect coupling process of C and N metabolism by the succession of microbes. Additionally, our study was conducted in coastal eutrophic regions, and the occurrence of some processes was region-specific (e.g., microbial populations responded quickly to SOM and there were obvious microbe-derived N-cycle processes) (Zheng et al., 2019). We observed the transformation of different forms of inorganic N over the incubations, however, the absence of organic nitrogen measurement limits further understanding about the flux budget of N.

## Conclusion

Our results clearly demonstrated that SOM could be quickly utilized by microbes and microbial-mediated processes of SOM metabolism also drove the elemental cycling of C, N, and P. Moreover, the quick utilization of FDOM component (e.g., C2), the generation and re-utilization of FDOM (e.g., C3) and the production of recalcitrant FDOM (e.g., C4) by microbial activity indicated that microbes could consume and transform SOM, affecting the bioavailability of DOM in the environment. Additionally, the variations in the microbial communities corresponded with the bioavailable TOC/SOM (phases I, II, and III) in the long-term incubations, implying the possible functional diversity coupling with environmental nutrient conditions. The different dominant microbial communities were present in different phases during SOM degradation which suggested different bioavailable substrates provide distinct ecological habitats suitable for specialized populations. The three identified phases, based on organic carbon consumption rates, corresponded with the accumulation processes of NH_4_^+^, NO_2_^–^, and NO_3_^–^, as well as the presence of dominant Thaumarchaeota and detectable levels of Nitrospinae/Nitrospirae in the later period showing an excellent coupling pattern between C and N metabolism.

The coupling of carbon and nitrogen metabolic processes mediated by coastal microbes during the degradation of SOM provided greater insight into the biogeochemical cycles in the eutrophic environment. Additionally, the bacterial community succession and degradation of organic matter in the long-term shed light on the vertical transformation processes of POM from euphotic zone to the darker and deeper parts of the ocean. Future work should be focused on determining the necessity of applying advance mass spectrometry technologies (e.g., Fourier transform ion cyclotron resonance mass spectrometry), in combination with metagenome and metatranscriptome analyses to strengthen our understanding of the microbial behaviors and microbial metabolites.

## Data Availability Statement

The datasets generated for this study can be found in the National Center for Biotechnology Information (NCBI) Sequence Read Archive (http://trace.ncbi.nlm.nih.gov/Traces/sra/) under BioProject PRJNA532855.

## Author Contributions

RX, QZ, and NJ conceived the study and designed the experiments. RX performed experiments and data analyses. YW undertook the 16S rDNA/rRNA analyses. QC assisted with the sampling. WG provided Varian Cary Eclipse spectro-fluorometer. RX and QZ wrote the manuscript. All authors reviewed the manuscript.

## Conflict of Interest

The authors declare that the research was conducted in the absence of any commercial or financial relationships that could be construed as a potential conflict of interest.

## Footnotes

1 http://ccb.jhu.edu/software/FLASH/

2 http://drive5.com/uparse/

3 http://trace.ncbi.nlm.nih.gov/Traces/sra/
