# Supplementary material for: Coupling Between Carbon and Nitrogen Metabolic Processes Mediated by Coastal Microbes in Synechococcus-Derived Organic Matter Addition Incubations
Source: Front Microbiol. 2020 May 25;11:1041. doi: 10.3389/fmicb.2020.01041 (PMC7261836; doi:10.3389/fmicb.2020.01041)
Supplement: Supplementary file 1 [file Data_Sheet_1.PDF]

SUPPORTING INFORMATION

Coupling between carbon and nitrogen metabolic processes mediated by coastal microbes in *Synechococcus*-derived organic matter addition incubations

The Supporting Information contains:

Table S1 Profile of the sampling stations

Table S2 Profile of the initial condition about two stations

Table S3 The relative contribution of specific OTUs to the Bray–Curtis index of dissimilarity

Figure S1 TOC and DOC concentration variations over the incubations (A, S05; B, S03). The inside figures showed the TOC and DOC variations in SOM-addition groups during the phase I.

Figure S2 Variations of inorganic nitrogen (NH_4^+, NO_2^- and NO_3^-) concentration during the entire incubation at station (A) S05 and (B) S03

Figure S3 Variation of PO_4^{3-} concentration during the entire incubation at station (A) S05 and (B) S03.

Figure S4 Microbial community composition based on 16S rDNA gene sequences at station S05 and S03 throughout the incubations, shown at the phylum level (except for Proteobacteria, which are divided into classes). Top: S05; down: S03. C-: control; F-: 0.22−3 μm size fraction (free-living fraction); P-: >3 μm size fraction (particle-associated fraction).

Figure S5 Correlation of TOC concentration variation between NH_4^+ and PO_4^{3-} concentrations variation in the phase I. S05: square; S03: triangle.

Figure S6 Nonmetric multidimensional scaling (NMDS) ordination based on Bray-Curtis similarities between microbial communities at station S05 and S03. Top: rDNA-based; down: rRNA-based. (A), (B): station S05; (C), (D): station S03. Each dot represents an individual sample in the NMDS figures. C-: control; F-: 0.22−3 μm size fraction (free-living fraction); and P-: >3μm size fraction (particle-associated fraction). Phase I: indicated by the green shading; Phase II: indicated by the gray shading; and Phase III: indicated by the blue shading.

Figure S7 Bacterial community variations in the Alphaproteobacteria at station S05 (A), (B) and S03 (C), (D). Top: rDNA-based; down: rRNA-based.

Figure S8 Bacterial community variations in the Gammaproteobacteria at station S05 (A), (B) and S03 (C), (D). Top: rDNA-based; down: rRNA-based.

Figure S9 Bacterial community variations in the Bacteroidetes at station S05 (A), (B) and S03 (C), (D). Top: Top: rDNA-based; down: rRNA-based.

Table S1 Profile of the sampling stations

Station	Northern Latitude	East longitude	Temperature (°C)	Salinity(psu)
S03	24°25.8424	118°02.3272	28.10	25.51
S05	24°25.1812	118°08.7784	27.80	28.74

Table S2 Profile of the initial nutrient conditions

Station	TOC(μM)	$NH4^+$(μM)	NO_2^-(μM)	NO_3^-(μM)	PO_4^{3-}(μM)
S03	98.43	8.44	2.79	48.42	1.00
S05	85.24	4.70	2.04	26.14	0.59

The data was from the controls at time zero.

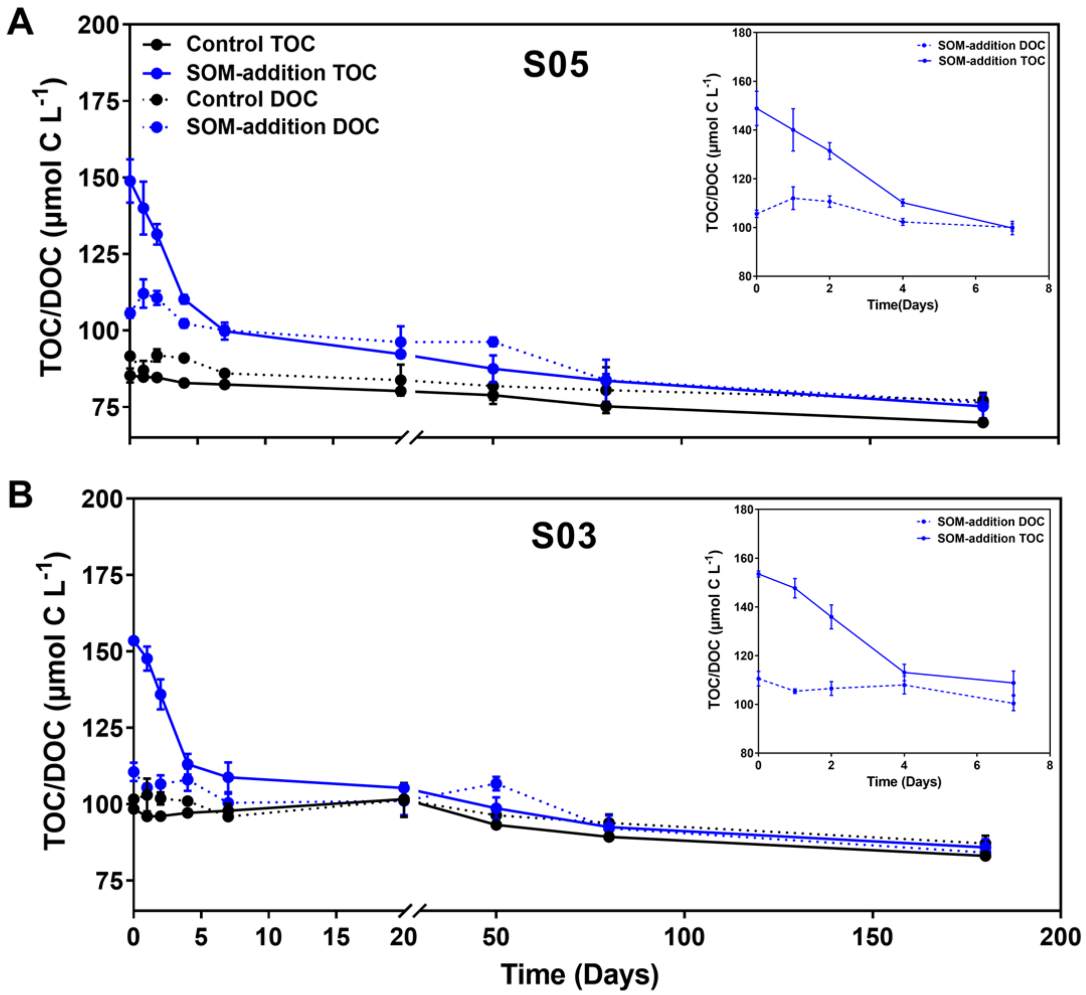

Figure S1 TOC and DOC concentration variations over the incubations (A, S05; B, S03). The inside figures showed the TOC and DOC variations in SOM-addition groups during the phase I.

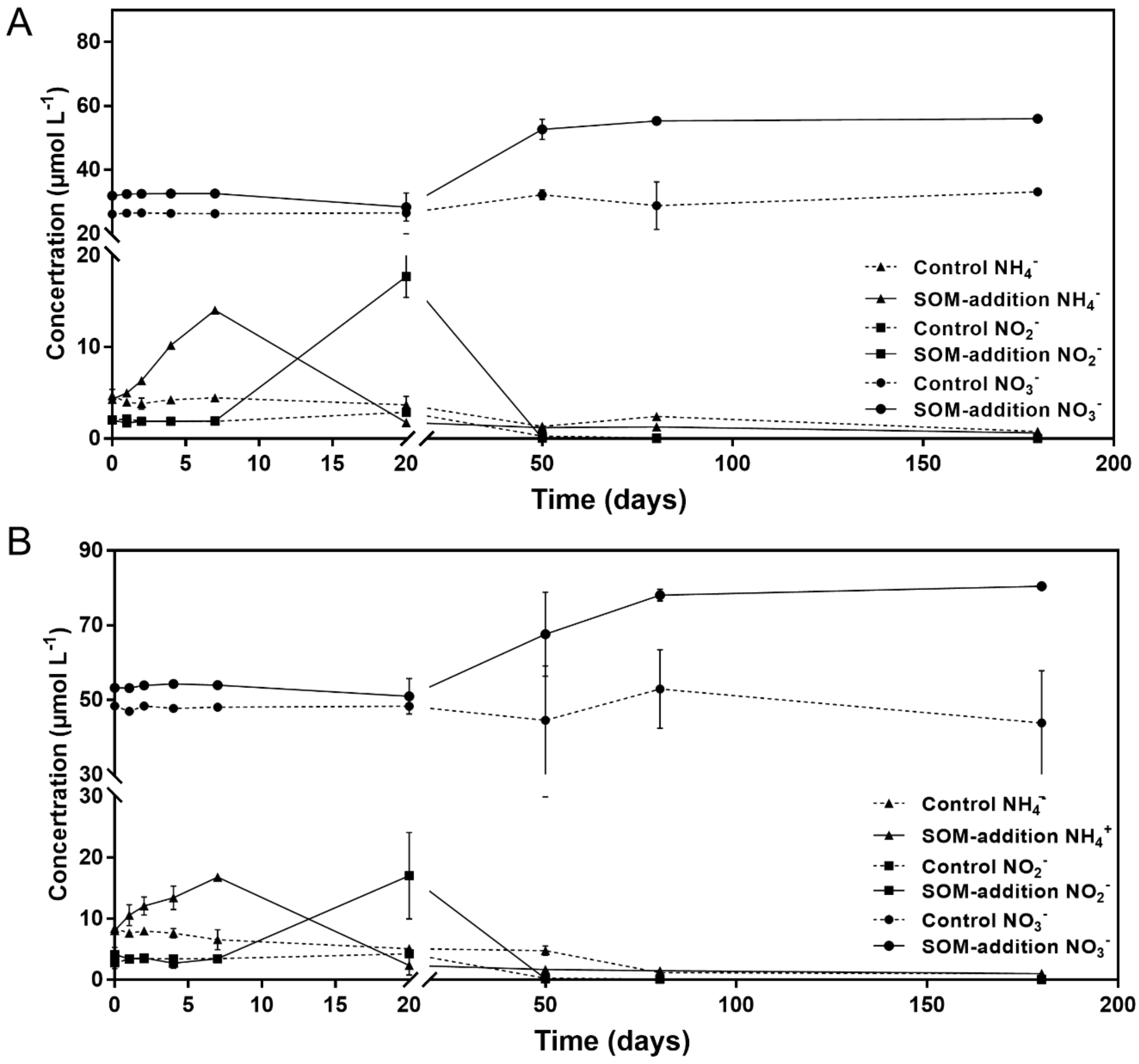

Figure S2 Variations of inorganic nitrogen (NH_4^+, NO_2^- and NO_3^-) concentration during the entire incubations at station (A) S05 and (B) S03.

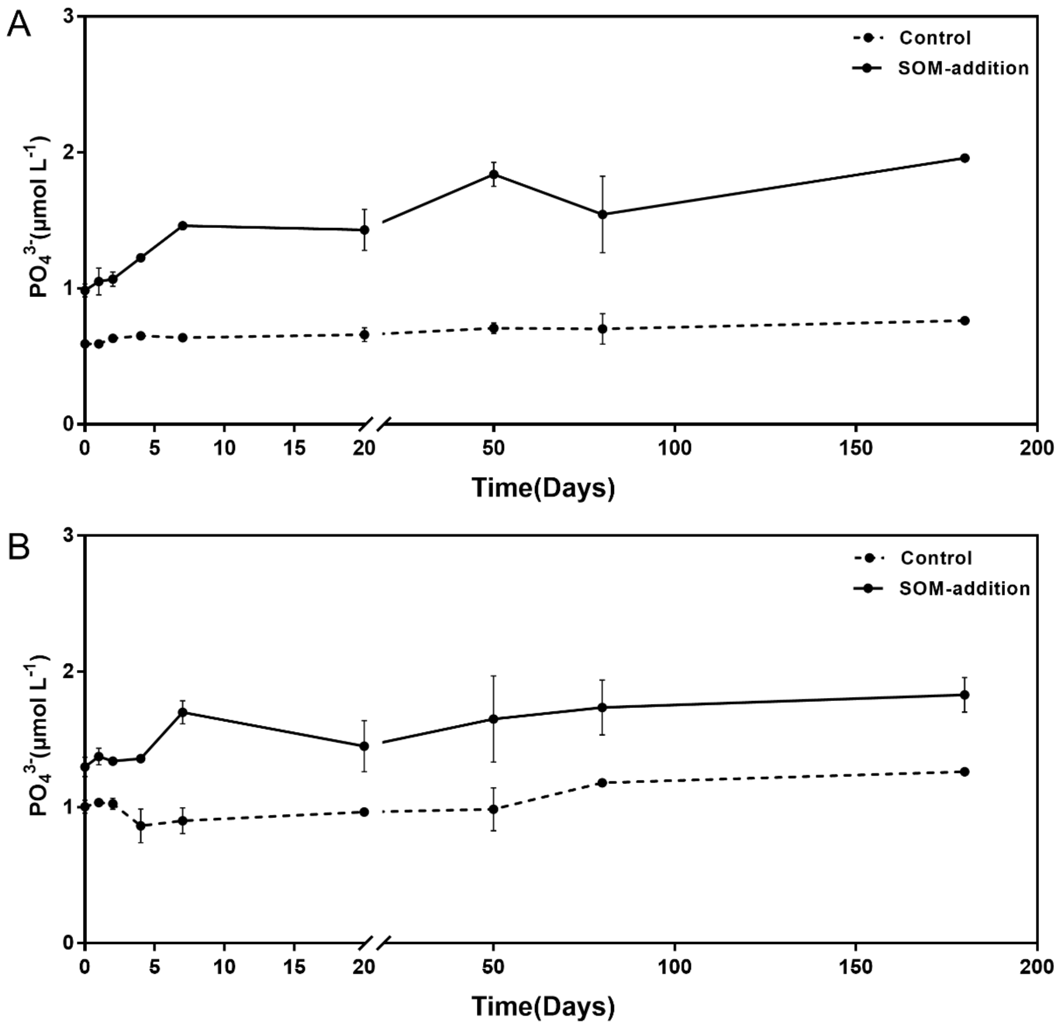

Figure S3 Variation of PO_4^{3-} concentration during the entire incubation at station (A) S05 and (B) S03.

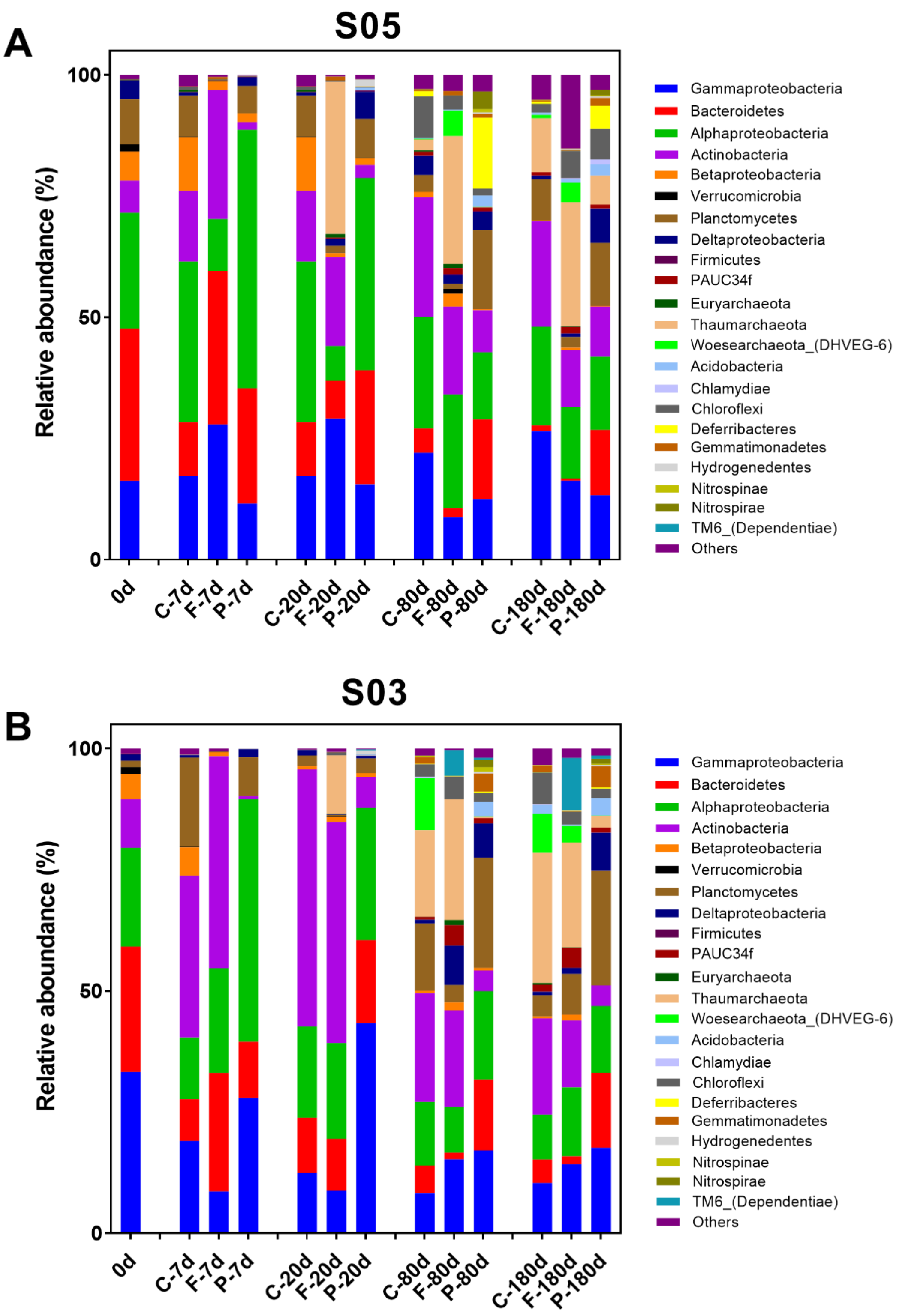

Figure S4 Microbial community composition based on 16S rDNA gene sequences at station (A) S05 and (B) S03 throughout the incubations, shown at the phylum level (except for Proteobacteria, which are divided into classes). Top: S05; down: S03. C-: control; F-: 0.22-3μm size fraction (free-living fraction); and P-: >3μm size fraction (particle-associated fraction).

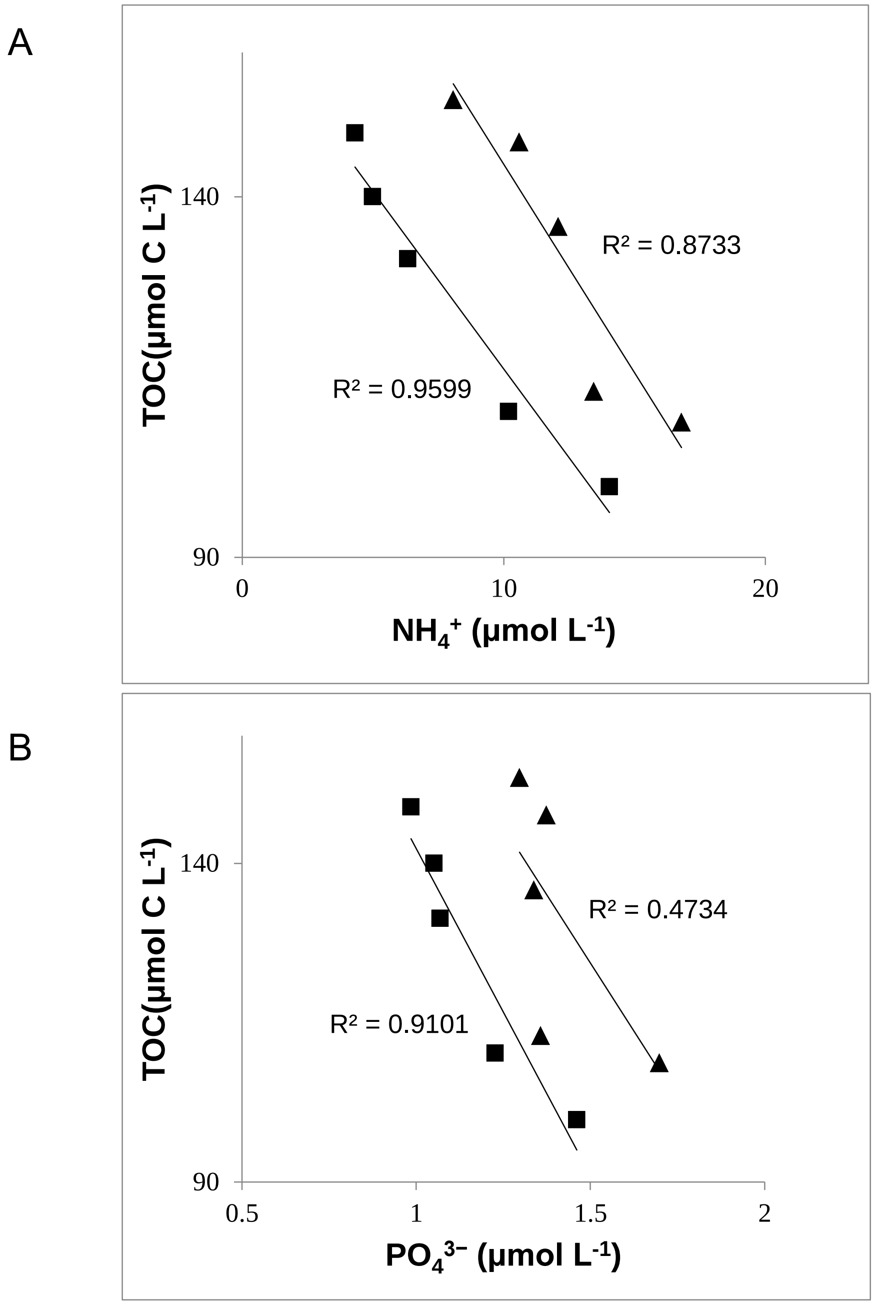

Figure S5 Correlation of TOC concentration variation between NH_4^+ (A) and PO_4^{3-} (B) concentrations variation in the phase I (day 0-7). S05: square; S03: triangle.

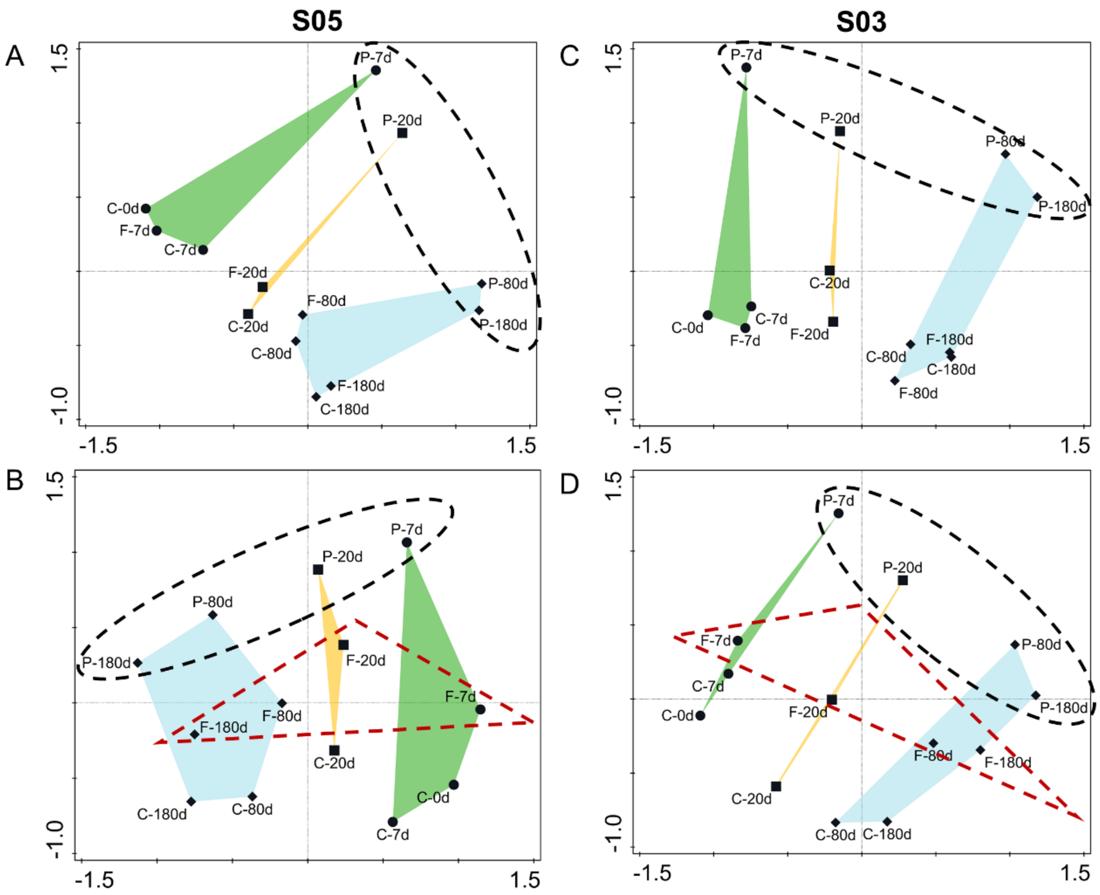

Figure S6 Nonmetric multidimensional scaling (NMDS) ordination based on Bray-Curtis similarities between microbial communities at station S05 and S03. Top: rDNA-based; down: rRNA-based. (A), (B): station S05; (C), (D): station S03. Each dot represents an individual sample in the NMDS figures. C-: control; F-: 0.22–3μm size fraction (free-living fraction); and P-: >3μm size fraction (particle-associated fraction). Phase I: indicated by the green shading; Phase II: indicated by the yellow shading; and Phase III: indicated by the blue shading.

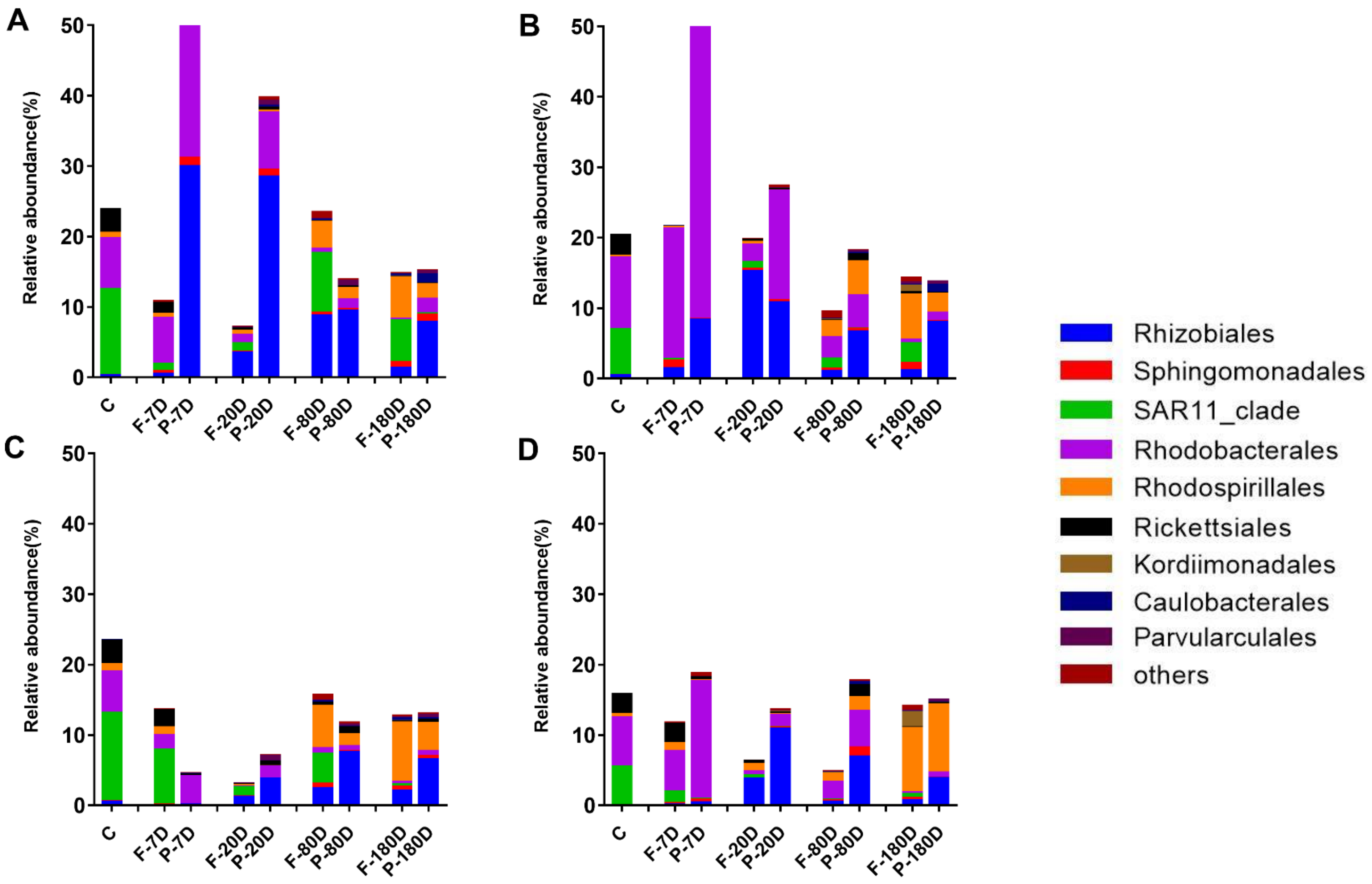

Figure S7 Bacterial community variations in the Alphaproteobacteria at station S05 (A), (B) and S03 (C), (D). Top: rDNA-based; down: rRNA-based.

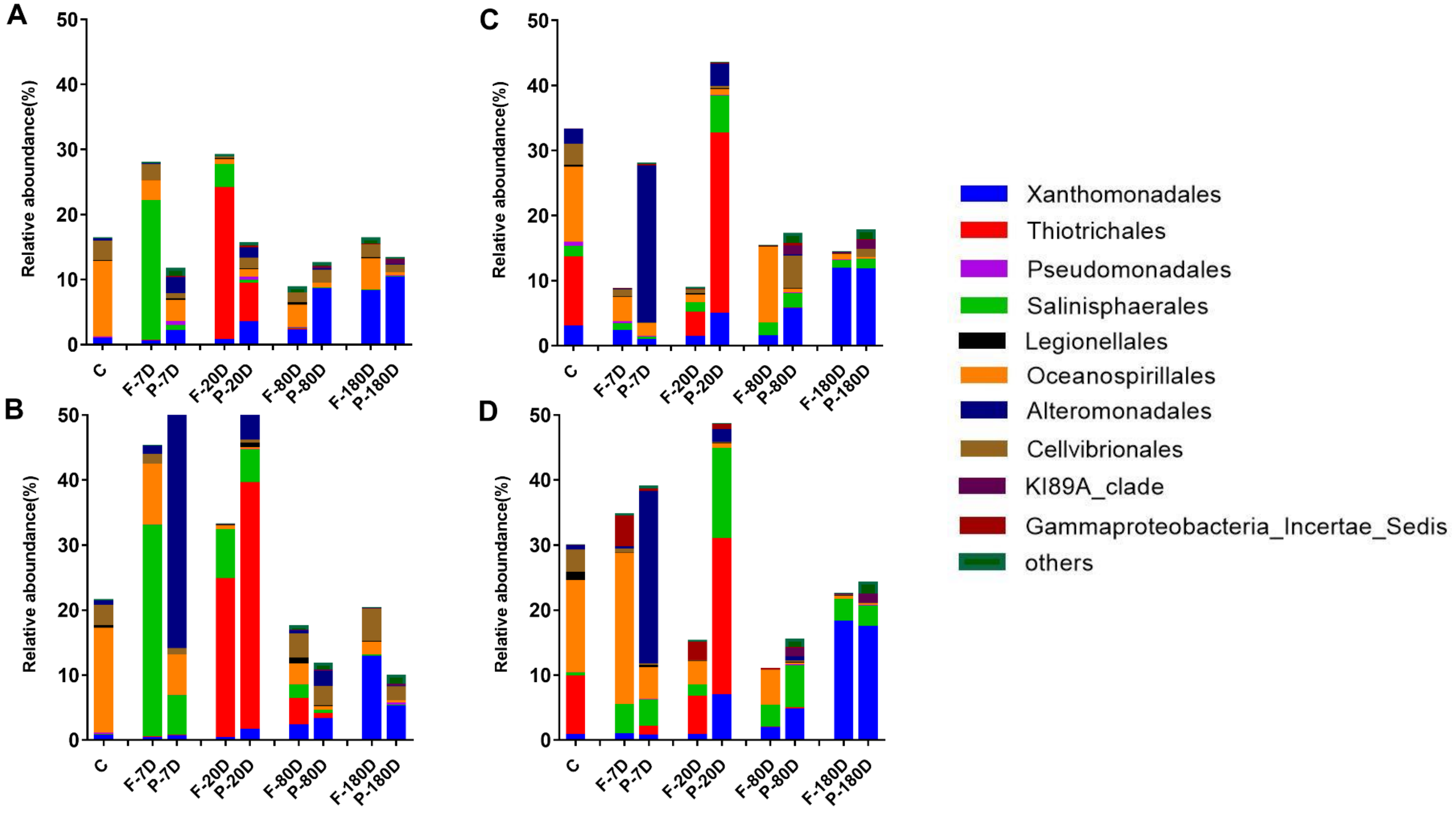

Figure S8 Bacterial community variations in the Gammaproteobacteria at station S05 (A), (B) and S03 (C), (D). Top: rDNA-based; down: rRNA-based.

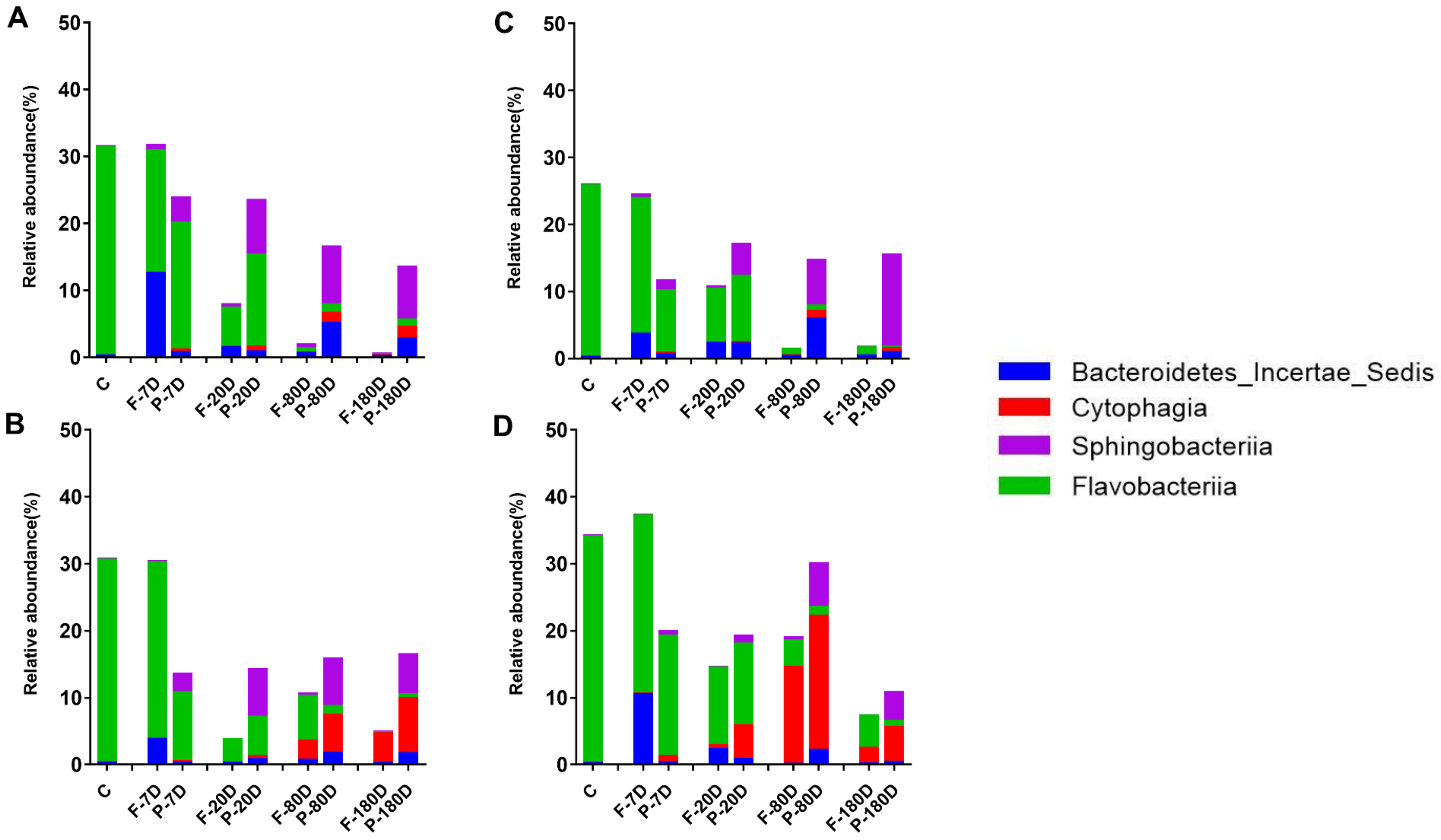

Figure S9 Bacterial community variations in the Bacteroidetes at station S05 (A), (B) and S03 (C), (D). Top: rDNA-based; down: rRNA-based.
